# Supplementary material for: Hippocampal pattern separation supports reinforcement learning
Source: Nat Commun. 2019 Mar 6;10:1073. doi: 10.1038/s41467-019-08998-1 (PMC6403348; doi:10.1038/s41467-019-08998-1)
Supplement: Supplementary file 1 — Supplementary Information [file 41467_2019_8998_MOESM1_ESM.docx]

**Supplementary Information**

*Hippocampal pattern separation supports reinforcement learning*

Ballard et. al

*SUPPLEMENTARY FIGURES*

*
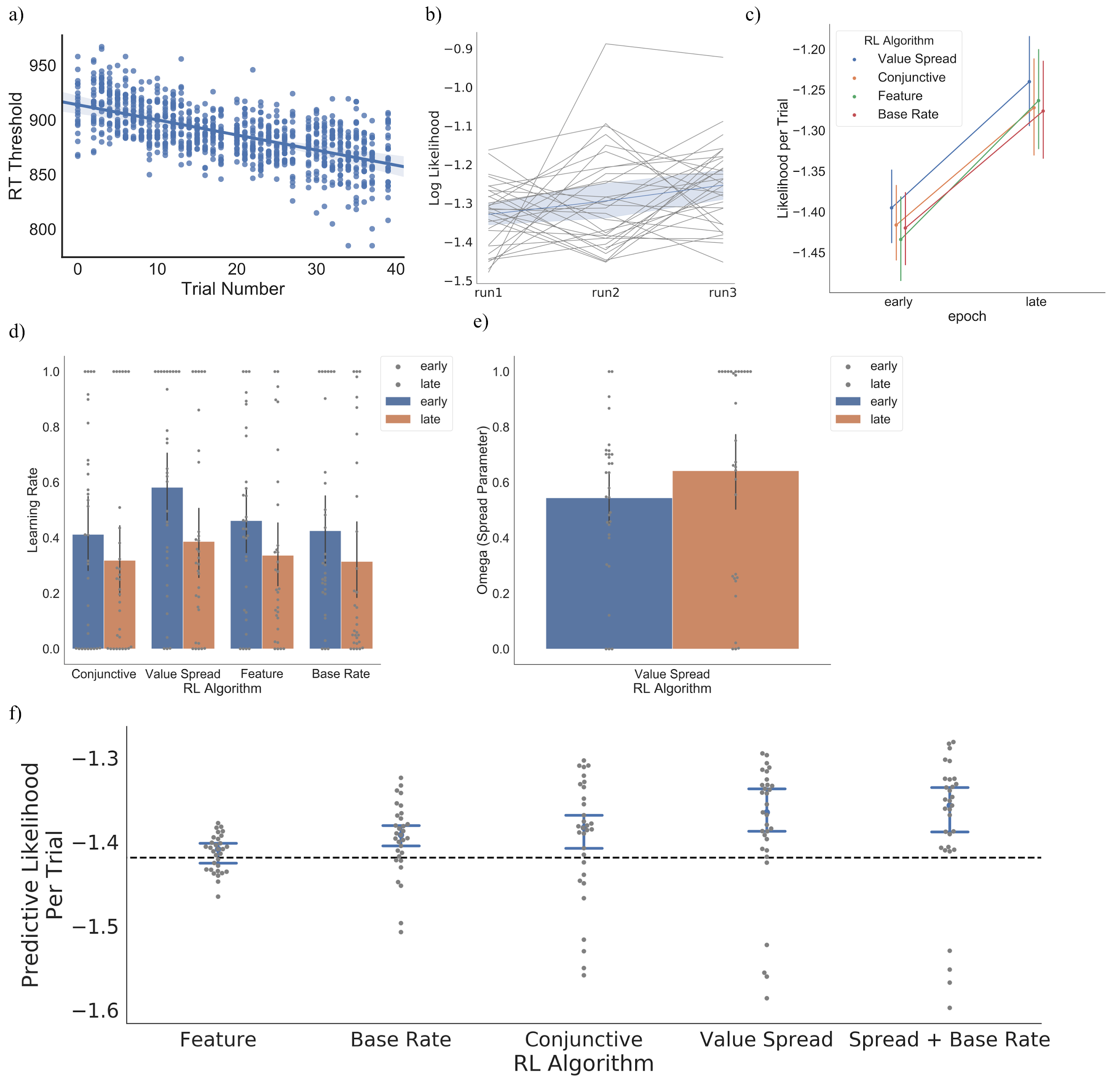
*

*Supplementary Figure 1. Supplemental behavioral data. Related to Figure 2. All error bars represent standard error of the mean. Source data are provided as a Source Data file.*

A) Reaction time threshold as a function of trial number. Each dot represents a subject. Subjects became gradually faster for AB+ and C+ trials over the course of each run, *Z* = -2.04, *p* = .041, mixed effect model with random intercepts for subjects. Accordingly, the response-based thresholding algorithm set a progressively more stringent threshold over the course of the run.

B) Likelihood for each run under the Value Spread RL model with maximum likelihood estimated parameters. Individual lines correspond to subjects. The run likelihood has been normalized to likelihood-per-choice. Whereas blocking or carry-over effects would reduce our model’s performance for runs 2 and 3 relative to run 1, the data show a small, but significant improvement in model-fit across runs, Z = 2.12, *p* = .034, mixed effects model.

C) Likelihood for each epoch, averaged across runs, with separate learning rate and value spread parameters for each epoch. We found an overall effect of epoch, *Z* = 4.7, *p* < .001, such that behavior was better fit by the models for later trials. However, we found no evidence of an interaction of model type (Feature versus Conjunctive) by epoch (early versus late), p > .2. This difference in fit across epochs could arise because conjunctive and feature representations were better crystalized later in learning, or simply because there were more unmodeled psychological effects on reaction times early in learning.

D) Learning rates estimated for each epoch. Note that fits to these parameters are often noisy at the individual-subject level. We find a main effect of reduced learning rate late in learning, *F*(1,30) = 6.11, *p* = .02, *n^2^* = .03, but no main effect of model, *p* = .12, nor any interaction between epoch and model. Thus, subjects more heavily weight prediction errors early in learning, when they are not sure which stimuli lead to the target; but later in learning, when it is more clear which stimuli are target-predictive, they weight prediction errors less.

E) Value spread parameter estimated for each epoch. We found no difference between epochs, *p* > .2.

F) Predictive log-likelihood per-trial for each of the models in the main text, as well as a Base Rate learning model and a Value Spread + Base Rate model. The Base Rate, Value Spread and Value Spread + Base Rate model all explain similar amounts of variance in unseen data. Importantly, the parameters of the Value Spread + Base Rate model indicate that both processes contribute to the variance explained (see Main Text).

Error bars for all panels depict bootstrapped estimates of the standard error of the group mean.


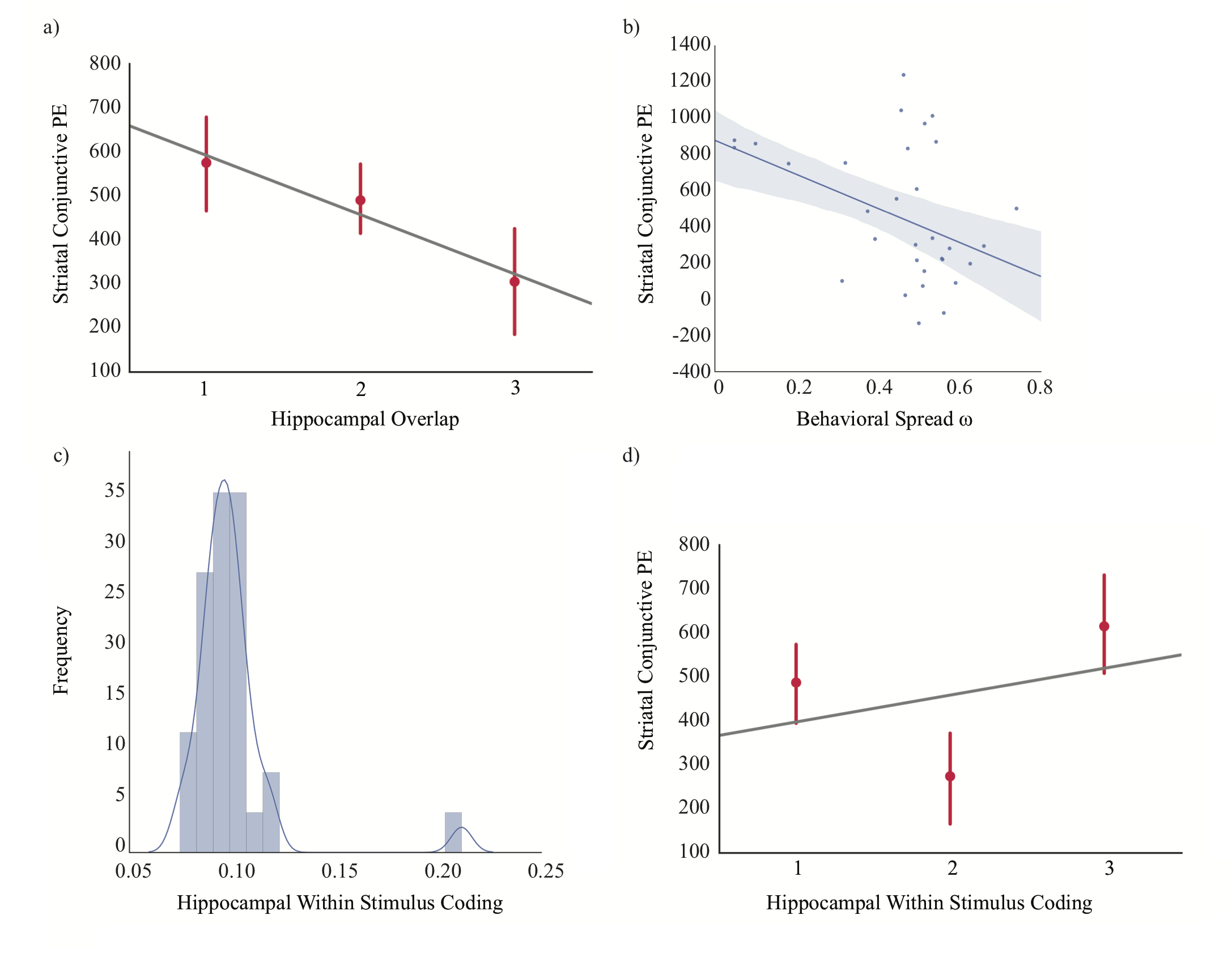


*Supplementary Figure 2. Relationship between neural and behavioral measures of pattern separation with the conjunctive component of the striatal prediction error. Related to Figures 3 and 4. Source data are provided as a Source Data file.* All error bars represent standard error of the mean.

A) Relationship between hippocampal pattern overlap and the conjunctive component of striatal prediction error. Data on the x-axis are regression coefficients for each run, organized from smallest to largest for each subject. Specifically, the labels [1,2,3] correspond to each of the 3 runs, ordered by the magnitude of hippocampal within-stimulus coding on that run. Data on the y-axis are regression coefficients extracted from the leave-one-subject-out striatal masks. For runs with more pattern overlap (i.e., lower pattern separation) between stimuli sharing features, the striatal prediction error response more closely tracks a model that confuses stimuli with overlapping features.

B) Subjects who showed stronger behavioral evidence of spreading value between stimuli with overlapping features (ω) from the Value Spread RL model) showed a reduced conjunctive component of the striatal prediction error. “Behavioral spread” is the degree to which value updates occurred for stimuli with overlapping features, as assessed from behavior. The plot depicts a robust partial correlation and the variance attributable to model-fit has been removed from the x-axis.

C) Distribution of betas on the within-stimulus similarity term of the regression model. Three outlier runs are from a single subject and are 4.9 standard deviations from the mean.

D) Relationship between hippocampal within-stimulus coding and the conjunctive component of striatal prediction error. Data on the x-axis are regression coefficients for each run, organized from smallest to largest for each subject. Data on the y-axis are regression coefficients extracted from the leave-one-subject-out striatal masks. We ran a mixed-effects model with subject as a random intercept and no random slope because the model failed to converge when within-stimulus coding was included as a random slope. For runs with stronger within-stimulus coding, the striatal prediction error response more closely tracked a model with the true, conjunctive state space, *t*(31) = 2.49, *p* = .013, *d_z_* = 0.45. Plot and statistic exclude outlier subject from C.

Error bars for all panels depict bootstrapped estimates of the standard error of the group mean.


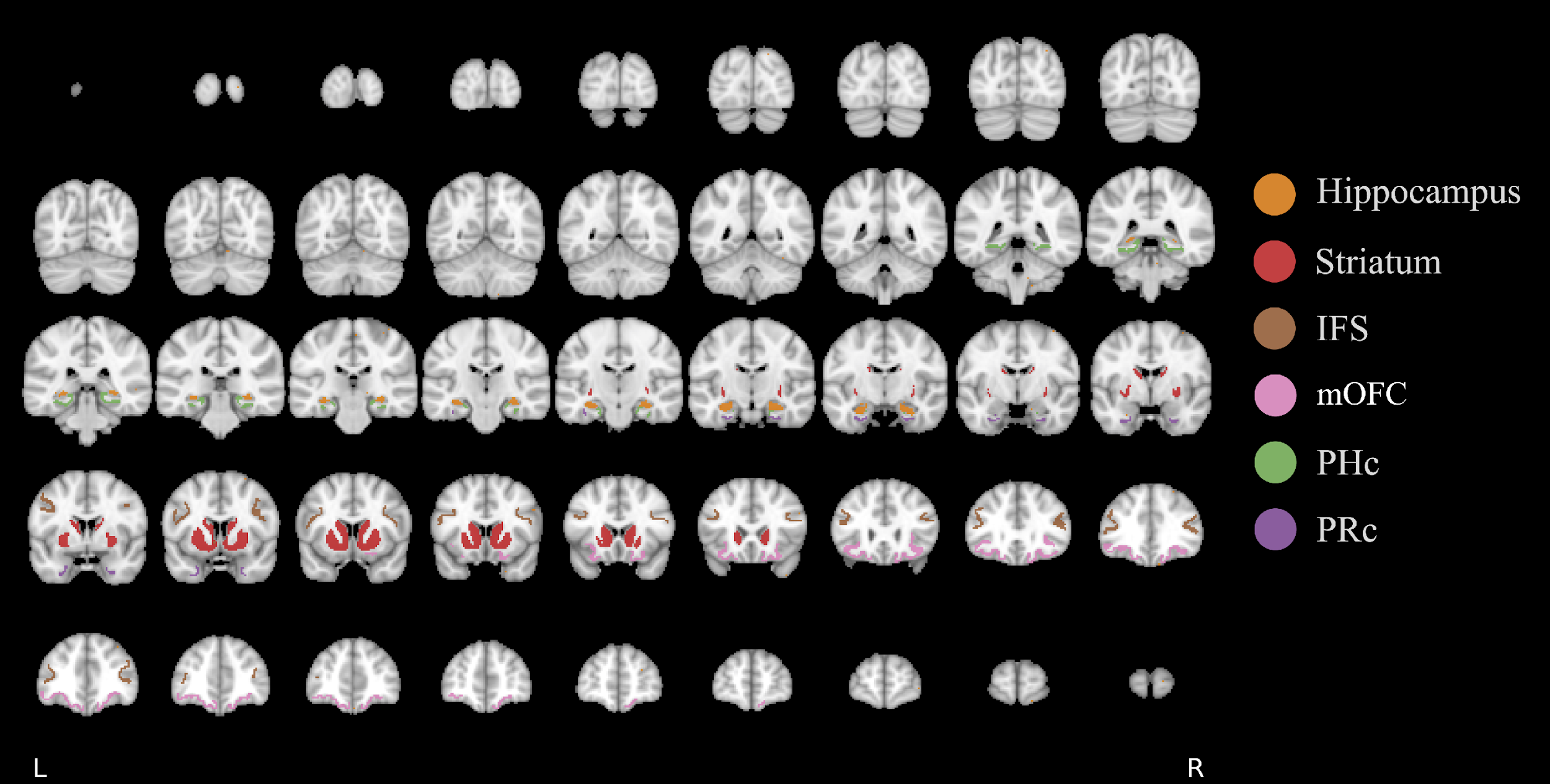


*Supplementary Figure 3: Depiction of the ROIs on the MNI152 brain. Related to Figures 3-6.*

Note that the striatal anatomical ROI (depicted here) was also crossed with the leave-one-subject-out functional Feature prediction error regressor mask.


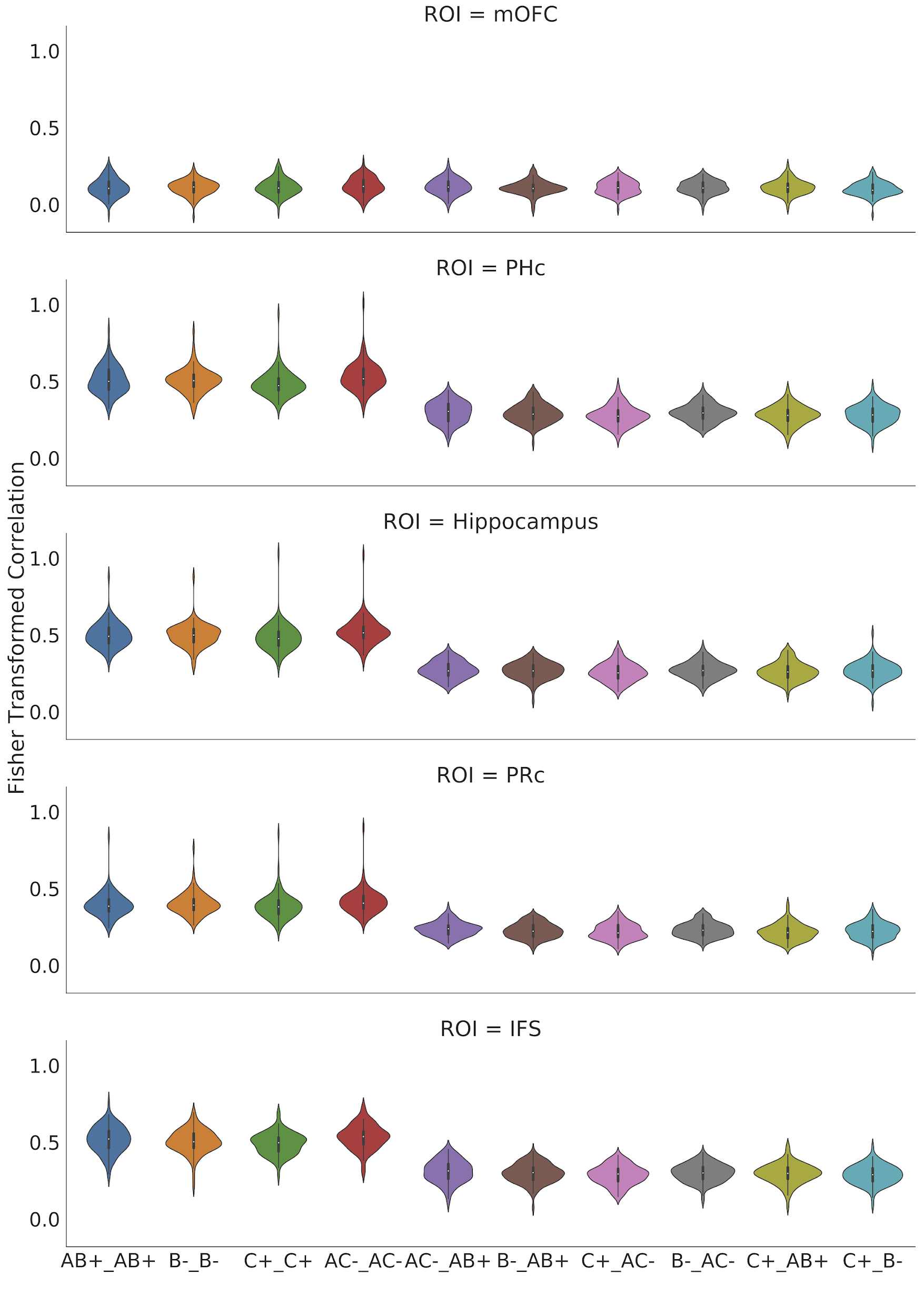


*Supplementary Figure 4: Depiction of similarity values for each comparison and ROI, related to Figures 4 and 5. Source data are provided as a Source Data file.* Note that the absolute magnitude of the correlations on the y-axis are not directly interpretable because they have been artificially inflated by within-run correlation. The effect of stimulus identity is readily apparent from the raw correlations. For between-stimulus comparisons, it is necessary to separate out the multiple contributors to pattern similarity via the regression approach described in the manuscript.

*
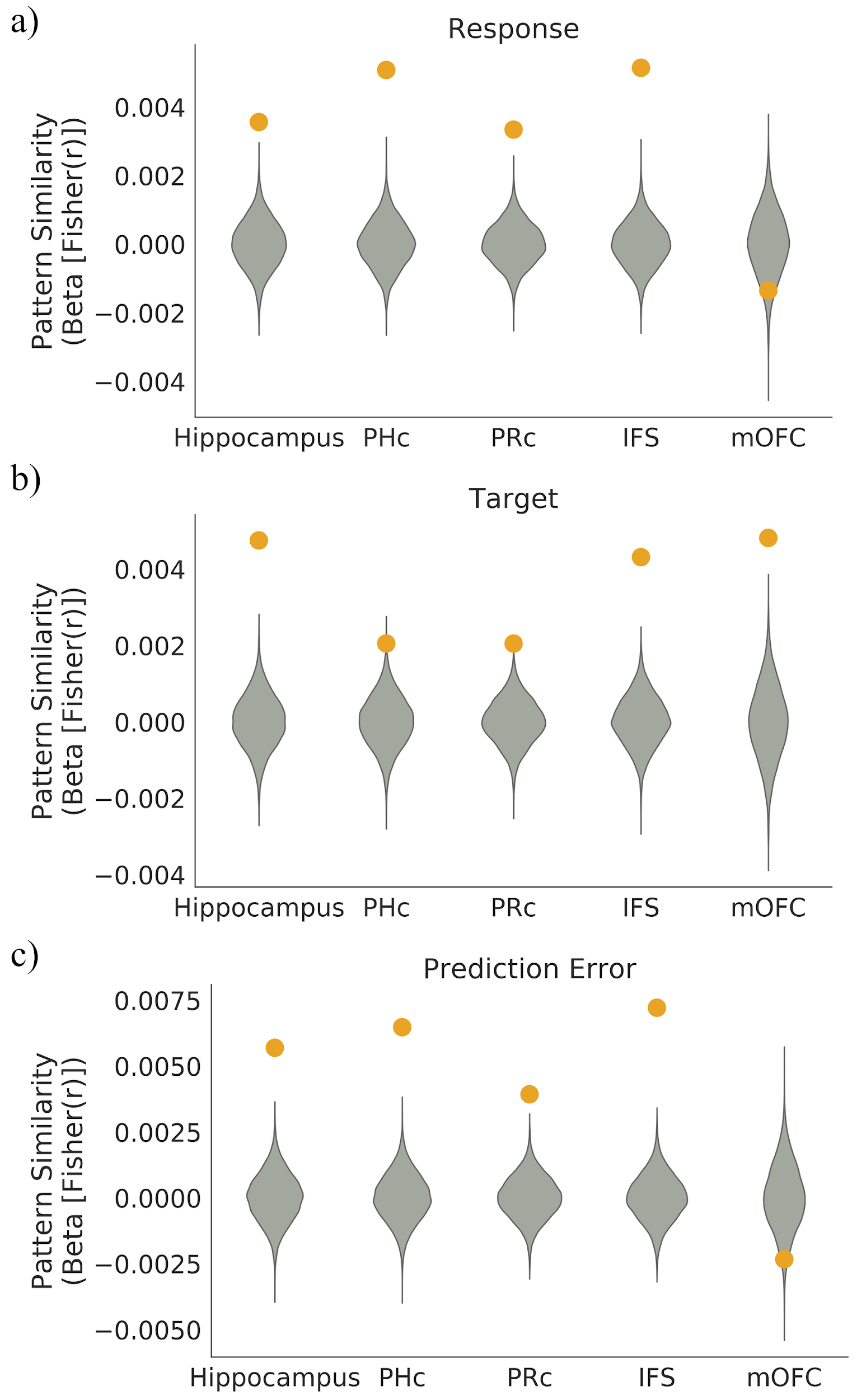
*

*Supplementary Figure 5. Analyses of other regressors on pattern similarity. Related to Figure 3. Source data are provided as a Source Data file.*

A) This analysis tests for similarity between stimuli that share a response (either both lead to target or both lead to non-target). This effect is positive in all ROIs, all *p* < .001, except the mOFC, which shows a trend in the opposite direct, *p* = .072, FDR corrected.

B) This analysis codes for additional similarity above and beyond the response similarity caused by pairs of stimuli where the target occurs. This effect is positive in all ROIs, PRc, *p* = .001, all other *p* < .001, FDR corrected. Together with A, this finding suggests that the mOFC patterns are primarily driven by the occurrence of the target.

C) Relationship between prediction error and pattern similarity. Trials with similar magnitudes of prediction error show higher pattern similarity across all ROIs, all *p* < .001, except mOFC, where stimuli are more dissimilar, *p* = .029, FDR corrected. The positive relationships likely reflect expectancy violation, which is associated with a univariate effect of the absolute value of prediction error in our data[^1^](https://paperpile.com/c/Z1wM0v/08Z3).


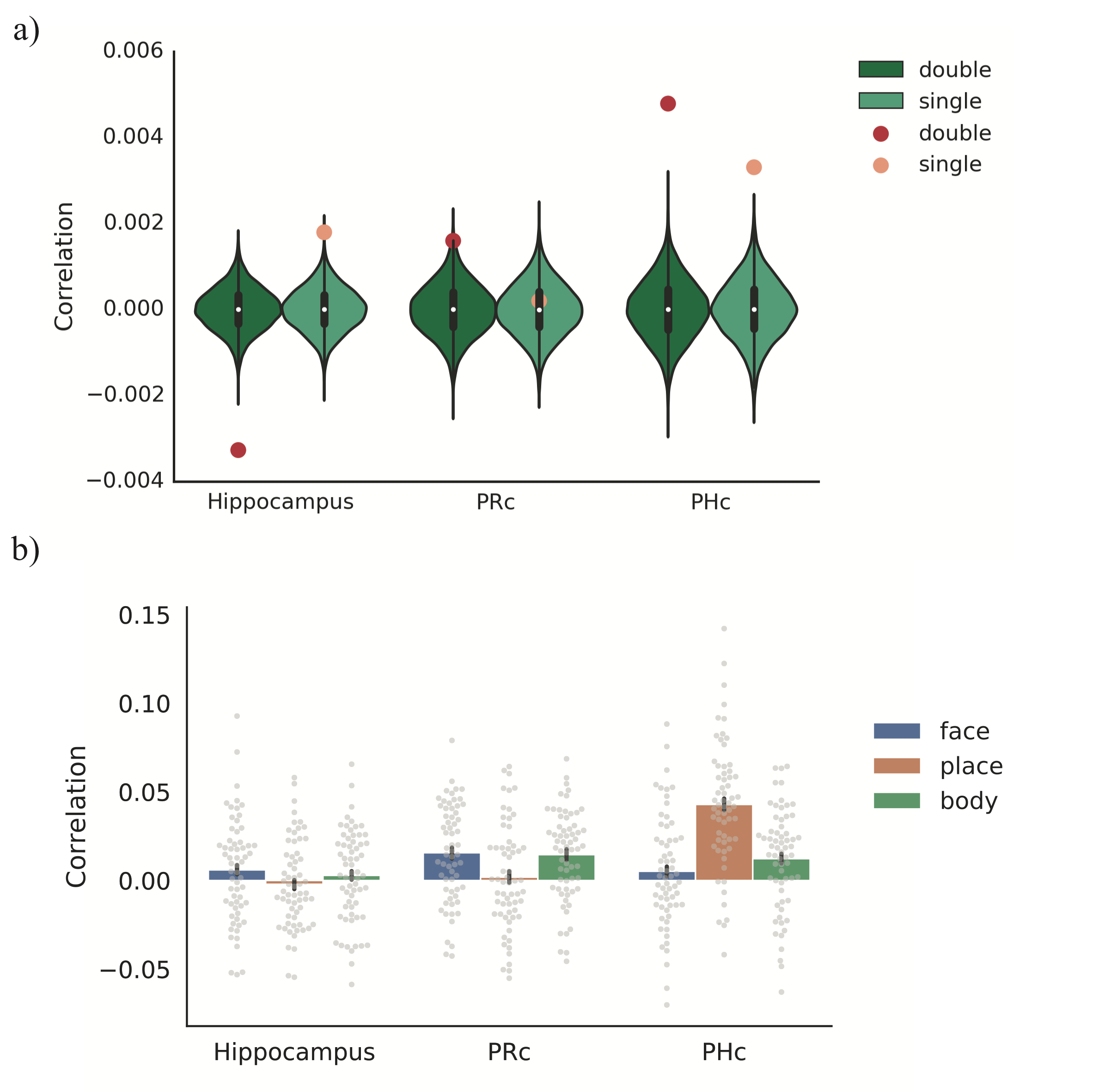
*Supplementary Figure 6. Control analyses of pattern content analysis. Related to Figure 6. Source data are provided as a Source Data file.*

A) Pattern content analysis with the effect of baseline subtracted shows that our results are not driven by stimulus-general activation. Note that this analysis underestimates the size of the correlation because it subtracts out the correlation with the stimulus category not present on the current trial; because we used a fast event-related design, this correlation is on average positive.

B) Correlation between main task trials and localizer category templates as a function of the category of the template, collapsing across single- and two-feature stimuli. We find stronger place similarity in PHc and stronger face and body part coding in PRc, consistent with the known category selectivity of these regions. Error bars depict bootstrapped estimates of the standard error of the group mean. Dots correspond to individual run means with the subject intercept removed.

|  | Mean | SD |
| --- | --- | --- |
| ω | .46 | .24 |
| α | .48 | .30 |
| β | -1.73 | 2.72 |

*Supplementary Table 1.* *Parameter means and standard deviations from the Value Spread RL model. Related to Figure 1. Source data are provided as a Source Data file*

*SUPPLEMENTARY NOTES*

*Supplementary Note 1: Value spreading versus mixed predictions.* We note that mixed conjunctive and feature learning could also arise if the predictions of independent feature and conjunctive learnings systems were mixed at the time of outcome prediction. However, we were unable to reliability fit such a model to our data. Therefore, we cannot adjudicate between the spreading of value updates during learning versus mixed predictions of independent systems. Nonetheless, both models share the core feature that subjects formed conjunctive representations of multi-featural stimuli but also learned about individual stimulus features.

*Supplementary Note 2: Multiple systems for learning.* It has been proposed that subjects rely on hippocampal learning early in probabilistic reward tasks, and then transfer to using striatal learning over time[^3^](https://paperpile.com/c/Z1wM0v/JW2Oo). We ran an additional control analysis to ensure that the performance of the Value Spread model across all trials did not reflect a transfer of learning from pure Conjunctive to pure Feature learning. We binned trials into early and late epochs, collapsing across runs. We examined the likelihood of the data for each epoch under the maximum likelihood parameter estimates for each model, Supplementary Figure S1. We found an overall effect of epoch, *Z* = 4.7, *p* < .001, mixed model, such that behavior was better fit by the models for later trials. However, we found no evidence of an interaction of model type (Feature versus Conjunctive) by epoch (early versus late), *p* > .2, mixed model. These results indicate that performance of the Value Spread model likely did not reflect a transition from Feature to Conjunctive learning.

*Supplementary Note 3: Base rate effects.* Response times are known to exhibit base rate effects: if many recent trials required a response, the response on the subsequent trial is likely to be faster[^2^](https://paperpile.com/c/Z1wM0v/ZyB5Q). Such effects could be misinterpreted as feature learning if temporally adjacent target trials happened to share features. Our task design mitigated this concern because different trial types were randomly intermixed. Nonetheless, we formally tested whether such base rate effects contributed to behavior. We constructed a Base Rate agent that learned the probability of a target, unconditional on the identity of the current stimulus. This agent outperformed chance, *T* = 98, *p* = .003, as well as the Feature learning model, *T* = 89, *p =* .002, and had no difference in predictive power from either the Conjunctive, *T* = 242, *p* > .2, or the Value Spread model, *T* = 172*, p* = .136, Wilcoxon tests (Supplementary Figure S1f). Because the Base Rate learning performed as well as the Value Spread model, it was important to assess whether the two models were capturing unique components of behavior. To do so, we constructed a combined Value Spread + Base Rate model in which values from both learners were entered as regressors on reaction time. The five parameters of this model, ω,$\alpha$*_Value Spread_*,$\beta$*_Value Spread_*, $\alpha$*_Base Rate_*,$\beta$*_Base Rate_*, were fit simultaneously. This addition did not significantly improve the likelihood of the Value Spread model, Wilcoxon *T* = 172, *p* = .14, Wilcoxon test. Importantly, the regression coefficients on both the Value Spread RL value estimate and the Base Rate values were negative and significant, Value Spread RL *T* = 84, *p* = .002; Base Rate RL *T* = 136, *p* = .028, Wilcoxon tests, indicating that higher values from both agents were each associated with faster reaction times. Fits to the spread parameter, $\omega$ (mean: .41), indicated a relatively strong mixing of Conjunctive and Feature learning, even when accounting for response perseveration effects. We concluded that base rate effects contributed to behavior but did not qualitatively influence performance of the Value Spread model.

*Supplementary Note 4: Between-subject correlation between learning and striatal PE*

In the main paper, we argued that striatal PE responses reflect a learning system with conjunctive knowledge. We tested whether individual variability in this neural measure of conjunctive learning was related to ω, our behavioral parameter that captures the extent to which learning was driven by conjunctions versus features. We regressed ω against the conjunctive PE BOLD effect extracted from the striatal ROI. When doing so, we included a nuisance regressor that indexed the degree to which the Value Spread model fit behavior. This nuisance regressor was necessary because ω is unconstrained for models that do not fit and tends to move towards extreme values when fits are poor. Between subjects, the strength of the conjunctive PE evident in BOLD responses was inversely related to the behavioral spread parameter ω, *t*(30) = -2.38, *p* = .024, *d_z_* = -0.43, Figure SB. We note that the magnitude of this relationship is potentially inflated because of our sample size[^4^](https://paperpile.com/c/Z1wM0v/wOiw). Because we were concerned about the influence of leverage points in our data, we confirmed this result using robust regression with the Huber loss function, *z*(30) = -2.21, *p* = .027. Therefore, to the extent that subjects showed behavior consistent with more selective learning about conjunctions (and thus lower learning over features), they showed stronger effect of conjunctive PE on striatal activation.

*Supplementary Note 5:* *The relationship between response and stimulus overlap.* There was a correlation between the overlap regressor and the effect of response, i.e., comparisons between stimuli with the same target outcome versus different target outcome, (*r* = .09). We included nuisance regressors to control for this effect. In addition, this correlation can only introduce a spurious increase in our overlap measure if cortical ROIs were more similar when the outcomes were different because there are more pairs with different outcomes in the non-overlap condition (i.e., [(AB+, AC-), (AB+, B-), (AC-, C+)] versus [(AB+, C+), (AC-, B-), (B-, C+)]). Empirically, the effect of the response was positive in all regions except mOFC, Supplementary Figure S5, indicating the residual effect of response coding not accounted for by our regression approach would lead to an underestimate in our main finding of interest.

*Supplementary Note 6: Control analysis of stimulus hemifield*

Our stimuli appeared randomly on either the left or right of the screen, and for two-feature stimuli, the left/right assignment of features varied randomly on each presentation. Because receptive fields become larger and more bilateral more anterior in the ventral visual stream, this raises the possibility that the differences between regions in within-stimulus coding might be influenced by a differing sensitivity to hemifield. Specifically, the hippocampus may have the most stable representations of stimuli simply because it may be the least sensitive to hemifield effects. To explore this possibility, we subsampled the PSA matrices by whether stimuli were presented in the same or in different hemifields. Hippocampus showed the strongest hemifield effect for both subsets of the data (all *p* < .001), suggesting that the within-stimulus similarity result is not driven by reduced sensitivity to hemifield.

We next tested whether hemifield influenced the hippocampal overlap result. For this test, we could not subsample trials with matched hemifield, because some key trials (e.g., B compared with C) never had the same stimulus in the same hemifield. Therefore, we subsampled cells where there was no feature presented in the same hemifield for either stimulus in the comparison. We found that the hippocampus showed no pattern overlap, *p* > .3, and demonstrated less overlap than PRc cortex, *p* = .028, PHc, *p* = .014, IFS, *p* = .003 and mOFC, *p* = .003, FDR corrected. These findings confirm that hemifield effects did not meaningfully impact the critical effects reported in the main text, potentially because attention shifts receptive fields in high-level visual cortex[^5^](https://paperpile.com/c/Z1wM0v/euWB).

*Supplementary Note 7: Mixed effects modeling of PSA matrices*

To verify our results with a different set of statistical assumptions, we fit a mixed effects linear model to the data with subject as a random intercept and random slopes for each ROI. Confirming the permutation results in the main text, we found an effect of within-stimulus similarity, *p* < .001, *Z* = 177, but no effect of overlap, *p* > .3, *Z*  = 0.18 in the hippocampus. Further, we found expected interactions between ROI and within-stimulus similarity in PRc, *p* < .001, *Z*  = -32.8, PHc, *p* < .001, *Z*  = -10.9, IFS, *p* < .001, *Z*  = -122, and mOFC, *p* < .001, *Z*  = -125. We also found expected interactions between ROI and overlap strength in PHc, *p* = .008, *Z*  = 2.65, IFS, *p* < .001, *Z*  = 6.1, and mOFC, *p* < .001, *Z*  = 3.86, and a trend in PRc, *p* = .094, *Z*  = 1.67.

*Supplementary Note 8: Between-subject correlation between hippocampal overlap and striatal PE*

In the main text, we reported a mixed effects analysis relating the magnitude of hippocampal pattern overlap across runs to the conjunctive component of the striatal PE. A test for a between-subjects correlation revealed a significant negative relationship between hippocampal pattern overlap and striatal conjunctive PE, *r*(30) = -.36, *p* = .04. The same caveat about effect size, given our sample size, is noted.

*Supplementary Note 9: Relationship between pattern overlap and striatal prediction error in cortical ROIs*

Given our finding that hippocampal overlap related to the conjunctive component of the striatal prediction error, we wondered whether this effect was specific to the hippocampus. We repeated the analysis for cortical ROIs and observed a similar relationship for PRc, *p* = .003, *Z =* -3.6, and PHc, *p* = .049, *Z =* -2.4, IFS, *p =* .045, *Z =* -1.9, but not mOFC, *p =* .12, *Z =* -1.5, FDR corrected. We re-ran this analysis with the difference in run-by-run likelihoods between the Value Spread and the Null model as a covariate to account for fluctuation in participant engagement. Random slopes for ROIs resulted in convergence errors and so we excluded them. We found similar results when accounting for run-by-run engagement differences in all ROIs: hippocampus, *p* = .036, *Z =* -2.4, PRc, *p* = .005, *Z =* -4.0, and PHc, *p* = .036, *Z =* -2.2, IFS, *p =* .036, *Z =* -2.4, but not mOFC, *p =* .18, *Z =* -1.3.

*Supplementary Note 10: Null results*

Although hippocampal signals were related to striatal prediction errors, and striatal errors were related to the degree of conjunctive-like measures of behavioral performance, we did not find that hippocampal pattern signals were significantly correlated with behavioral performance. However, we note that we have low power to detect even moderate-size correlations with our sample size. We fitted an additional reinforcement learning model that adjusts the strength of the value leak between stimuli by the degree of pattern similarity between those stimuli, as measured from hippocampus activity. We were unable to reliably fit this model to individual subjects, which precluded Bayesian analysis. Even so, this model did not perform better than the Value Spread model on our cross-validation measure. We speculate that hippocampal patterns are only indirectly related to behavior via their impact on the striatum, and therefore it is harder to detect these relationships with our sample size. In addition, we did not detect a relationship between our hippocampal content analysis and the main pattern similarity analysis. Because affine registration makes for imperfect alignment between localizer and task data, we expect the results of this analysis to be noisier than our PSA analysis and this noise may have overshadowed individual or intra-run differences.

*Supplementary Note 11: Mixed effects modeling of pattern content analysis*

We modeled the correlations with a mixed effects model with random intercepts for each subject and random slopes for the effect of each ROI. We found the expected interaction between ROI and pattern similarity in both PHc, *p* < .001, *Z* = -4.1, and PRc, *p* = .005, *Z* = -2.9, indicating that the hippocampus had reduced pattern similarity to templates relative to the cortical ROIs. Further, we found the expected interaction between single-vs-two-feature similarity and ROI for both PHc, *p* = .026, *Z* = -2.2, and PRc, *p* = .009, *Z* = -2.6, indicating that the hippocampus had a larger difference in similarity between single and double features than the cortical ROIs. We also conducted a planned test for whether the hippocampal pattern similarity was higher for single than for two-feature stimuli and found a significant effect, *p* = .033, *Z* = 2.1, indicating a gradient in pattern-separation as the number of features increases. Post-hoc tests of the same comparison in the cortex revealed a trend towards the opposite effect in PRc, *p* = .086, *Z* = -1.7, and no effect in PHc, *p* > .2.

*Supplementary Note 12: Category selectivity in pattern content analysis*

Our pattern content analysis depends on the ability to match patterns of cortical activity during the localizer task to those in the learning task. As an indirect assessment of our method, we examined whether we could detect known gradients in category content along the parahippocampal gyrus, spanning PRc and PHc[^6^](https://paperpile.com/c/Z1wM0v/O4AV). We replotted the data from Figure 4 according to whether the target localizer stimulus in the correlation was a place, face, or body part (Figure S4). We constructed mixed effects ANOVAs with deviation coding. These analyses revealed stronger similarity for place stimuli in PHc, *z* = 4.1*, p* < .001*,* and relatively more similarity for body, *z* = 3.0, *p* = .008, and face, *z* = 3.7, *p* < .001 stimuli in PRc, consistent with the known gradient of category selectivity between these two regions. In hippocampus, while content effects were markedly reduced[^7^](https://paperpile.com/c/Z1wM0v/YHc8), we found significantly increased similarity for faces, *z* = 3.4, *p* = .001.

*Supplementary Note 13: Control analysis of stimulus-general activation in the pattern content analysis*

For our pattern content analysis, we computed the correlation between the patterns elicited by each trial of the main task with template patterns acquired during the localizer task. One possible concern with this approach is that it detects similarities that are stimulus-general, rather than stimulus-specific. For example, if similarity between the task and localizer were driven by neural activity that is similar across all stimuli, the response to a face during the task would be equally similar to the response to a face or a house in the localizer. However, this possibility should not give rise to the between-ROI results we report in the main text. Nonetheless, we investigated this directly by subtracting from each correlation a baseline correlation. We computed this as follows: Assume that A is a face, B is a house, and C is a body part. When looking at AB trials, we want to assess its similarity to the house template. We can instead assess the relative similarity between {face and house} and {face} to the similarity between {face and house} and {body part}. That is, we construct a baseline from the category not present in the main comparison. This baseline is imperfect because the trials occurred relatively close in time and there were lingering effects of the other stimulus category. However, other potential baselines are less suitable. Although our localizer data included object trials and written character trials, we cannot use the object templates because the target was a racecar, nor can we use a written character template, as subjects were alerted to the start of a new trial with text.

After subtracting out baseline correlations, we observed an overall similar pattern of results (Figure S4). We report the results from a mixed-effects model. We found the expected interaction between ROI and pattern similarity in both PHc, *p* < .001, *Z* = -4.1, and PRc, *p* = .034, *Z* = -2.1, indicating that the hippocampus had reduced pattern similarity to templates relative to the cortical ROIs. Further, we found the expected interaction between single-vs-two-feature similarity and ROI for both PHc, *p* = .017, *Z* = -2.4, and PRc, *p* = .018, *Z* = -2.4, indicating that the hippocampus had a larger difference in similarity between single and double features than the cortical ROIs. We also conducted a planned test for whether the hippocampal pattern similarity was higher for single than for two-feature stimuli and found a significant effect, *p* = .004, *Z* = 2.9, consistent with a gradient in pattern-separation as the number of features increase. Post-hoc tests of the same comparison in the cortex revealed no effect in PRc, *p* > .2 nor in PHc, *p* > .2. We therefore exclude stimulus-general activation as an account of our effects.

*SUPPLEMENTARY DISCUSSION*

We contend the hippocampus forms representations of conjunctions of features that are reinforced via dopamine release on hippocampal-striatal synapses, but the hippocampus could form a representation of the temporal sequence of task events[^8^](https://paperpile.com/c/Z1wM0v/Uo6dJ). In AB+ trials, AB could trigger a representation of the target in the hippocampus, and this target representation could then feed into the striatum or prefrontal cortex to drive responses. This model is similar to the idea that the hippocampus encodes a “successor representation” for reinforcement learning[^9^](https://paperpile.com/c/Z1wM0v/PFCpV) in which the target representation occurs in proportion to the probability of each stimulus preceding the target. The hippocampus-to-striatum connectivity and successor representation explanations of our results differ in mechanism but share the requirement of a conjunctive representation in the hippocampus. Future work should directly test the role of hippocampal sequence representation in reinforcement learning.

*SUPPLEMENTARY METHODS*

| RESOURCE | SOURCE |
| --- | --- |
| FSL 5.0.8 | https://fsl.fmrib.ox.ac.uk/fsl/fslwiki |
| ANTs 1.9 |  |
| Lyman 0.0.10 | http://www.cns.nyu.edu/~mwaskom/software/lyman/ |
| Freesurfer 5.3.0 | https://surfer.nmr.mgh.harvard.edu/ |
| R 3.3.1 | https://www.r-project.org/ |
| IFS definition | https://surfer.nmr.mgh.harvard.edu/fswiki/CorticalParcellation_Yeo2011 |

*Supplementary Table 2: Key resource table.*

*Localizer task information*

After completing all three runs of the experiment, subjects completed two runs of a task designed to localize visual category selective regions of cortex. The localizer stimuli included presentations of body parts (limbs or full bodies without heads), objects (cars or guitars), faces (adult or child), characters (nonsense words and strings of numbers), places (places or houses), or a fixation cross. Stimuli were shown in miniblocks of 4 s at a rate of 2 Hz. To balance the stimuli on low-level visual features, stimuli were presented against a background that was constructed from a phase-scrambled image from a different visual category. Subjects were instructed to respond if they saw a phase-scrambled image without a recognizable stimulus overlaid.

*Model comparison procedures*

To compare different models, we used two complementary approaches with different underlying assumptions. The first, a leave-one-rule-out cross-validation approach, provides a useful null hypothesis statistical test (NHST) and penalizes over-complex models because of their lack of generalizability to unseen data[^10^](https://paperpile.com/c/Z1wM0v/4Fp4). The second, a Bayesian approach, is based on an estimate of the log-model evidence, which includes a penalty for complex models. Unlike the NHST, the Bayesian approach provides an easily interpretable metric of the probability that each model generated the observed data. For the Bayesian comparison procedure, each model was fit to each subject, and the maximum likelihood estimates were used to compute the corrected Akaike Information Criterion (AIC) as an estimate of the log-model evidence. These were then submitted to a random effects Bayesian model selection procedure that is sensitive to outliers[^11^](https://paperpile.com/c/Z1wM0v/MQgs). This procedure provides the probability, above and beyond chance, that a randomly chosen subject would be best explained by each model. For the NHST approach, we implemented a leave-one-subject-out approach. For each fold, each RL model was fit to the data of the remaining subjects. The model, along with the maximum a posteriori parameter estimates, was then used to predict reaction times of the held-out subject. Leave-one-subject out modeling provides beneficial regularization over a leave-one-run-out within-subject approach, resulting in improved predictive accuracy[^12^](https://paperpile.com/c/Z1wM0v/oyiq). Note that in cases where we analyzed parameters of the models, such as significance testing on the beta relating values to reaction times, we computed these parameters independently for each subject.

*MRI Acquisition*

Imaging was performed on a 3.0 Tesla GE Discovery MR750 scanner. High-resolution T1-weighted scans were acquired using an MP-RAGE sequence. We acquired multiplexed functional data with a multiband factor of 3. This allowed us to acquire partial brain data covering the frontal, temporal, and occipital lobes at high spatial resolution (1.6 × 1.6 × 1.6 mm). Functional acquisition details were as follows: echo-planar imaging, interleaved acquisition, gradient recalled echo; TR = 1500 ms; TE = 30 ms; flip angle = 77°.

*fMRI Preprocessing*

Analyses were conducted using tools from FSL, Freesurfer and ANTs, implemented using the Lyman fMRI analysis software, http://www.cns.nyu.edu/~mwaskom/software/lyman/. High-resolution anatomical images were segmented into grey and white matter components with Freesurfer, and a model of the cortical surface was computed. Functional data were aligned to the middle slice using sinc interpolation. We acquired additional volumes with an opposing phase encoding gradient direction that allowed us, using the FSL TOPUP tool, to infer and correct for magnetic field inhomogeneity due to susceptibility artifacts. We next implemented a noise-reduction procedure using FSLs FIX tool, which removes ICA-derived components of the data that are identified as noise by a publicly available classifier trained on independent data. Slices with artifacts were automatically identified as frames on which total displacement relative to the previous frame exceeded 0.5 mm or in which the average intensity across the whole brain deviated from the run mean by greater than three and a half standard deviations. Images were high-pass filtered by fitting and removing Gaussian-weighted running lines with an effective cycle cutoff of 128 s and were smoothed with a 4-mm full-width at half-maximum (FWHM) Gaussian kernel. Finally, images were co-registered to the anatomical data using Freesurfer’s boundary-based registration algorithm with six degrees of freedom.

*PSA Preprocessing*

For PSA, we extracted parameter estimates from each ROI and regularized these estimates with multivariate noise normalization[^13^](https://paperpile.com/c/Z1wM0v/drtK). To do this, we computed an estimate of the noise covariance from the residuals of the general linear models for each ROI. This matrix was then regularized using the optimal shrinkage parameter, inverted, and multiplied by the vector of betas for each condition. This approach removes nuisance correlations between voxels that arise due to physiological and instrument noise. Importantly, this transformation tends to increase correlations, but does not induce any bias between conditions. However, the magnitude of correlations should not be compared to different experiments. We next computed pairwise Pearson correlations between conditions for all 40 trials of each run. This approach is conceptually similar to using the Mahalanobis distance, but our approach provides a distance metric that is invariant to changes in the scale of patterns across conditions. We did not subtract the mean pattern across conditions, as this causes spurious anti-correlations. We computed within-run, rather than between-run correlations. We did this because stimuli-to-label mappings were counterbalanced across runs (e.g., A could be a face in run one and a house in run two). We expected representations to form in each run that were idiosyncratic to the particular stimuli.

*Region of Interest Selection*

All ROIs are depicted in coronal slices in Figure S3. We used the Freesurfer segmentation to define the hippocampus, perirhinal, parahippocampal and medial orbitofrontal ROIs. We defined the IFS ROI from the prefrontal cognitive control component of a parcellation of the cortex into networks that show correlated resting-state activation reliably in a cohort of 1,000 subjects[^14,15^](https://paperpile.com/c/Z1wM0v/XJo9+A0Dp). This type of ROI is beneficial because it is more concretely defined than broad anatomical labels (e.g., dlPFC), and because resting-state based parcellation methods detect meaningful functional parcels of cortex[^16^](https://paperpile.com/c/Z1wM0v/wUXu). Further, feature-based rule learning and execution is generally associated with activation in the IFS[^12,17^](https://paperpile.com/c/Z1wM0v/oyiq+KjJZ). We did not use this procedure for the mOFC because resting-state based parcellations are less reliable in areas of signal dropout. To create our cortical ROIs, we warped region labels back to the individual subject surfaces by inverting the spherical normalization parameters obtained during cortical reconstruction. Vertex coordinates within each of these labels were then transformed into the native functional space by inverting the linear functional-to-anatomical transformation for the first run. Finally, ROI masks were constructed by projecting half the distance of the cortical thickness at each vertex and labeling the intersected voxels.

For the striatal ROI analysis, we constructed an ROI from a joint functional and anatomical mask. We used an executive-limbic striatal ROI taken from a 3-way subdivision of striatum based on diffusion tractography imaging estimated connectivity with cortex[^18^](https://paperpile.com/c/Z1wM0v/2QFr). This relatively large subdivision includes most of the anterior striatum. For each subject, we crossed this anatomical ROI with a functional mask created from the feature prediction error map constructed from a group analysis of the other subjects and thresholded at *p* < .05 uncorrected. Because the conjunctive prediction error regressor was constructed as a difference, it is only interpretable in voxels that have a feature prediction error response. The leave-one-subject-out functional ROI approach allows for examination of a region that is sensitive to feature prediction error while avoiding reverse inference.

*SUPPLEMENTARY REFERENCES*

1. [Davis, T., Xue, G., Love, B. C., Preston, A. R. & Poldrack, R. A. Global neural pattern similarity as a common basis for categorization and recognition memory. *J. Neurosci.* **34**, 7472–7484 (2014).](http://paperpile.com/b/Z1wM0v/08Z3)

2. [Lau, B. & Glimcher, P. W. Dynamic response-by-response models of matching behavior in rhesus monkeys. *J. Exp. Anal. Behav.* **84**, 555–579 (2005).](http://paperpile.com/b/Z1wM0v/ZyB5Q)

3. [Poldrack, R. A. *et al.* Interactive memory systems in the human brain. *Nature* **414**, 546 (2001).](http://paperpile.com/b/Z1wM0v/JW2Oo)

4. [Yarkoni, T. Big Correlations in Little Studies: Inflated fMRI Correlations Reflect Low Statistical Power—Commentary on Vul et al. (2009). *Perspect. Psychol. Sci.* **4**, 294–298 (2009).](http://paperpile.com/b/Z1wM0v/wOiw)

5. [Kay, K. N., Weiner, K. S. & Grill-Spector, K. Attention Reduces Spatial Uncertainty in Human Ventral Temporal Cortex. *Curr. Biol.* **25**, 595–600 (2015).](http://paperpile.com/b/Z1wM0v/euWB)

6. [Davachi, L. Item, context and relational episodic encoding in humans. *Curr. Opin. Neurobiol.* **16**, 693–700 (2006).](http://paperpile.com/b/Z1wM0v/O4AV)

7. [Liang, J. C., Wagner, A. D. & Preston, A. R. Content Representation in the Human Medial Temporal Lobe. *Cereb. Cortex* **23**, 80–96 (2013).](http://paperpile.com/b/Z1wM0v/YHc8)

8. [Gluck, M. A. & Myers, C. E. Hippocampal mediation of stimulus representation: A computational theory. *Hippocampus* **3**, 491–516 (1993).](http://paperpile.com/b/Z1wM0v/Uo6dJ)

9. [Stachenfeld, K. L., Botvinick, M. M. & Gershman, S. J. The hippocampus as a predictive map. *Nat. Neurosci.* **20**, 1643 (2017).](http://paperpile.com/b/Z1wM0v/PFCpV)

10. [Niv, Y. *et al.* Reinforcement learning in multidimensional environments relies on attention mechanisms. *J. Neurosci.* **35**, 8145–8157 (2015).](http://paperpile.com/b/Z1wM0v/4Fp4)

11. [Rigoux, L., Stephan, K. E., Friston, K. J. & Daunizeau, J. Bayesian model selection for group studies - revisited. *Neuroimage* **84**, 971–985 (2014).](http://paperpile.com/b/Z1wM0v/MQgs)

12. [Ballard, I., Miller, E. M., Piantadosi, S. T., Goodman, N. D. & McClure, S. M. Beyond Reward Prediction Errors: Human Striatum Updates Rule Values During Learning. *Cereb. Cortex* **19**, 1–11 (2017).](http://paperpile.com/b/Z1wM0v/oyiq)

13. [Walther, A. *et al.* Reliability of dissimilarity measures for multi-voxel pattern analysis. *Neuroimage* **137**, 188–200 (2016).](http://paperpile.com/b/Z1wM0v/drtK)

14. [Waskom, M. L., Frank, M. C. & Wagner, A. D. Adaptive Engagement of Cognitive Control in Context-Dependent Decision Making. *Cereb. Cortex* **27**, 1270–1284 (2017).](http://paperpile.com/b/Z1wM0v/XJo9)

15. [Choi, E. Y., Yeo, B. T. T. & Buckner, R. L. The organization of the human striatum estimated by intrinsic functional connectivity. *J. Neurophysiol.* **108**, 2242–2263 (2012).](http://paperpile.com/b/Z1wM0v/A0Dp)

16. [Glasser, M. F., Coalson, T., Robinson, E. & Hacker, C. A Multi-modal parcellation of human cerebral cortex. *Nature* (2015).](http://paperpile.com/b/Z1wM0v/wUXu)

17. [Badre, D. & D’Esposito, M. Is the rostro-caudal axis of the frontal lobe hierarchical? *Nat. Rev. Neurosci.* **10**, 659–669 (2009).](http://paperpile.com/b/Z1wM0v/KjJZ)

18. [Tziortzi, A. C. *et al.* Connectivity-Based Functional Analysis of Dopamine Release in the Striatum Using Diffusion-Weighted MRI and Positron Emission Tomography. *Cereb. Cortex* **24**, bhs397–1177 (2013).](http://paperpile.com/b/Z1wM0v/2QFr)
