# Supplementary material for: Hippocampal pattern separation supports reinforcement learning
Source: Nat Commun. 2019 Mar 6;10:1073. doi: 10.1038/s41467-019-08998-1 (PMC6403348; doi:10.1038/s41467-019-08998-1)
Supplement: Supplementary file 3 — Reporting Summary [file 41467_2019_8998_MOESM3_ESM.pdf]

natureresearch

Corresponding author(s): Ian Ballard

Last updated by author(s): Feb 7, 2019

Reporting Summary

Nature Research wishes to improve the reproducibility of the work that we publish. This form provides structure for consistency and transparency in reporting. For further information on Nature Research policies, see Authors & Referees and the Editorial Policy Checklist.

Statistics

For all statistical analyses, confirm that the following items are present in the figure legend, table legend, main text, or Methods section.

n/a	Confirmed	
☐	☒	The exact sample size (n) for each experimental group/condition, given as a discrete number and unit of measurement
☐	☒	A statement on whether measurements were taken from distinct samples or whether the same sample was measured repeatedly
☐	☒	The statistical test(s) used AND whether they are one- or two-sided<br>*Only common tests should be described solely by name; describe more complex techniques in the Methods section.*
☐	☒	A description of all covariates tested
☐	☒	A description of any assumptions or corrections, such as tests of normality and adjustment for multiple comparisons
☐	☒	A full description of the statistical parameters including central tendency (e.g. means) or other basic estimates (e.g. regression coefficient) AND variation (e.g. standard deviation) or associated estimates of uncertainty (e.g. confidence intervals)
☐	☒	For null hypothesis testing, the test statistic (e.g. F, t, r) with confidence intervals, effect sizes, degrees of freedom and P value noted<br>*Give P values as exact values whenever suitable.*
☒	☐	For Bayesian analysis, information on the choice of priors and Markov chain Monte Carlo settings
☒	☐	For hierarchical and complex designs, identification of the appropriate level for tests and full reporting of outcomes
☐	☒	Estimates of effect sizes (e.g. Cohen's d, Pearson's r), indicating how they were calculated

Our web collection on statistics for biologists contains articles on many of the points above.

Software and code

Policy information about availability of computer code

Data collection	Custom experimental code was used that is available online at https://github.com/iancballard/feature-discrimination/Hipp_Pattern_Separation_Code.
Data analysis	Custom analysis code is available at https://github.com/iancballard. I also used the following free software: FSL 5.0.8, ANTS 1.9, Lyman 0.0.10, Freesurfer 5.3.0 and R 3.3.1

For manuscripts utilizing custom algorithms or software that are central to the research but not yet described in published literature, software must be made available to editors/reviewers. We strongly encourage code deposition in a community repository (e.g. GitHub). See the Nature Research guidelines for submitting code & software for further information.

Data

Policy information about availability of data

All manuscripts must include a data availability statement. This statement should provide the following information, where applicable:

- Accession codes, unique identifiers, or web links for publicly available datasets
- A list of figures that have associated raw data
- A description of any restrictions on data availability

Raw MRI and behavioral data are available at OpenNeuro with accession code ds001590. Analysis code is available at https://github.com/iancballard/Hipp_Pattern_Separation_Code. The source data underlying Figs 2a–f, 3b, 4a-b, 5a-b, 6b and Supplementary Figs 1a-f, 2a-d, 4, 5a-c, 6a-b and Table 1 are provided as a Source Data file. Key resources are listed in Supplementary Table S2.

Field-specific reporting

Please select the one below that is the best fit for your research. If you are not sure, read the appropriate sections before making your selection.

☒ Life sciences ☐ Behavioural & social sciences ☐ Ecological, evolutionary & environmental sciences

For a reference copy of the document with all sections, see nature.com/documents/nr-reporting-summary-flat.pdf

Life sciences study design

All studies must disclose on these points even when the disclosure is negative.

Sample size	We did not perform an a priori power analysis. We collected data from 40 subjects hoping to get usable data from at least 30. This sample size is larger than is typical in the field of fMRI (around 15-20), and was therefore aimed to maximize experimental power above and beyond field standards while staying within the budget for our study.
Data exclusions	Data from eight subjects were excluded from analyses: One ended the scan early due to claustrophobia; three had scanner-related issues that prevented reconstruction or transfer of their data; two had repeated extreme (>2mm) head movements across most runs; and two subjects demonstrated extremely poor performance, as indexed by less than $2.50 of earnings (see below for payment details). Note that our task was calibrated to the individual subjects' practice data in such a way that a simple target detection strategy would be expected to earn $7.50, and any effort to learn the task should improve on these earnings.
Replication	We did not undertake any replication effort.
Randomization	Randomization of experimental conditions was applied within-subject
Blinding	We had no group allocations and therefore did not blind

Reporting for specific materials, systems and methods

We require information from authors about some types of materials, experimental systems and methods used in many studies. Here, indicate whether each material, system or method listed is relevant to your study. If you are not sure if a list item applies to your research, read the appropriate section before selecting a response.

Materials & experimental systems

n/a	Involved in the study	
☒	☐	Antibodies
☒	☐	Eukaryotic cell lines
☒	☐	Palaeontology
☒	☐	Animals and other organisms
☐	☒	Human research participants
☒	☐	Clinical data

Methods

n/a	Involved in the study	
☒	☐	ChIP-seq
☒	☐	Flow cytometry
☐	☒	MRI-based neuroimaging

Human research participants

Policy information about studies involving human research participants

Population characteristics	There were 32 subjects in the analysis cohort, 19 females, mean age 22.1 years, SD 3.14, range 18 to 29
Recruitment	Subjects were recruited through a participant sign-up website maintained by the Stanford Psychology Department. Subjects who were interested in the study could select it and sign up for timeslots. We do not see any way in which a selection bias could have influenced the results.
Ethics oversight	The study design and methods were approved by and followed the ethical procedures of the Stanford University Institutional Review Board

Note that full information on the approval of the study protocol must also be provided in the manuscript.

Magnetic resonance imaging

Experimental design

Design type	event-related

Design specifications	3 runs per subject. Each run contains 40 trials, 10 each of 4 different stimulus types (AB, AC, B, C). A, B, and C categories are balanced across the 3 runs. Inter-trial intervals and the interval between the stimulus/stimulus+target and feedback were taken from a Poisson distribution with a mean of 5 s, truncated to have a minimum of 2 s and maximum of 12 s. Because of the jitter and the fact that only trials with targets had feedback, the length of each trial was variable. Each block was roughly 11 minutes.
Behavioral performance measures	Behavioral performance was assessed as faster reaction times for stimuli that are predictive of the target. The predictiveness of target was parameterized using reinforcement learning models. We also used a model-free analysis of reaction time, hit rate and false alarms using random-effects ANOVAs.

Acquisition

Imaging type(s)	functional, structural
Field strength	3T
Sequence & imaging parameters	T1-weighted scans were acquired using an MP-RAGE sequence. We acquired multiplexed functional data with a multiband factor of 3. This allowed us to acquire at high spatial resolution (1.6 × 1.6 × 1.6 mm). Functional acquisition details were as follows: echo-planar imaging, interleaved acquisition, gradient recalled echo; TR = 1500 ms; TE = 30 ms; flip angle = 77∘.
Area of acquisition	partial brain data covering the frontal, temporal, and occipital lobes

Diffusion MRI ☐ Used ☒ Not used

Preprocessing

Preprocessing software	FSL 5.0.8, Freesurfer 5.30, ANTs 1.9, Lyman 0.0.10
Normalization	Volumetric data were normalized to the MNI template using ANTs 1.9 for whole-brain analysis. Cortical data was mapped to the freesurfer cortical template using Freesurfer 5.3.0. The cortical mapping was inverted in order to map cortical ROIs to native space and subsequent ROI analyses were conducted in native space
Normalization template	volumetric: MNI152, freesurfer: fsaverage
Noise and artifact removal	We implemented a noise-reduction procedure using FSLs FIX tool, which removes ICA-derived components of the data that are identified as noise by a publicly available classifier trained on independent data.
Volume censoring	Slices with artifacts were automatically identified as frames on which total displacement relative to the previous frame exceeded 0.5 mm or in which the average intensity across the whole brain deviated from the run mean by greater than three and a half standard deviations. This procedure was implemented by the Lyman software.

Statistical modeling & inference

Model type and settings	We used both univariate and PSA techniques. For the group univariate effects, we used mixed-effects modeling as implemented in FSL FLAME1. For PSA, we extracted parameter estimates separately for each run from a GLM that modeled each trial with a separate regressor. We performed regression on PSA matrices and evaluated the significance of these regression coefficients using both mixed effects modeling in R and nonparametric shuffling of RSA matrices. We used both statistical techniques to ensure that our results were robust to different statistical assumptions and we found convergent results from both statistical methods for all of our main results.
Effect(s) tested	We tested several effects:<br><br>1) We tested whether prediction errors taken from the feature learning model covaried with striatal BOLD in the whole-brain<br>2) We tested whether these same voxels had additional variance that could be explained by prediction errors from a learner that knows about conjunctions (the difference between the conjunctive and feature PEs) in a leave-one-subject-out functional ROI<br>3) We tested whether the hippocampus showed stronger pattern-similarity to repeated presentations of the same stimulus within a block than our control ROIs<br>4) We tested whether the hippocampus showed less pattern similarity between pairs of stimuli sharing features than our control ROIs<br>5) We tested whether the effect in 4 is related to the effect in 2 across runs. This tests whether runs with more pattern seperation in hippocampus are also runs with striatal errors with a higher contribution of conjunctive learning.<br>5) We tested whether the hippocampus showed reduced pattern similarity to feature pattern templates relative to our control ROIs<br>6) We tested whether the hippocampus showed reduced pattern similarity between stimuli with pairs of features (AB, and AC) and feature templates than between stimuli with single features (B and C) and feature templates.<br>7) We tested whether our ROIs showed increased or decreased representational distance as a function of the strength of association between stimuli and the target.

Specify type of analysis: ☐ Whole brain ☐ ROI-based ☒ Both

Anatomical location(s)	We used the Freesurfer segmentation to define the hippocampus, perirhinal, and parahippocampal ROIs. We defined the IFS ROI from the prefrontal cognitive control component of a parcellation of the cortex into networks that show correlated resting-state activation reliably in a cohort of 1,000 subjects (Waskom, Frank, & Wagner, 2017; Yeo et al., 2011).<br><br>For the striatal ROI analysis, we constructed an ROI from a joint functional and anatomical mask. We used an executive-limbic striatal ROI taken from a 3-way subdivision of striatum based on diffusion tractography imaging estimated connectivity with cortex (Tziortzi et al., 2013). This relatively large subdivision includes most of the anterior striatum. For each subject, we crossed this anatomical ROI with a functional mask created from the feature prediction error map constructed from a group analysis of the other subjects and thresholded at $p < .05$ uncorrected. Because the conjunctive prediction error regressor was constructed as a difference, it is only interpretable in voxels that have a feature prediction error response. The leave-one-subject-out functional ROI approach allows for examination of a region that is sensitive to feature prediction error while avoiding reverse inference.
Statistic type for inference<br>(See Eklund et al. 2016)	For the striatal feature PE regressor, we used FSLs FWE cluster GRF correction with the default cluster-defining threshold of z=2.3. All other analyses were ROI based and used both permutation-based statistics and mixed-effects modeling implemented in R.
Correction	We used FSL's standard FWE for the one whole-brain analysis. ROI analyses are FDR corrected across ROIs.

Models & analysis

n/a	Involved in the study
☒	☐ Functional and/or effective connectivity
☒	☐ Graph analysis
☐	☒ Multivariate modeling or predictive analysis

Multivariate modeling and predictive analysis	We used pattern similarity analysis on regression coefficients extracted from general linear models using the Pearson correlation coefficient. We reduced spatial autocorrelation in regression coefficients across voxels within an ROI using multivariate noise normalization based on the residuals on the GLM. We did not do any mean pattern subtraction, as this can induce bias in the PSA results (Garrido, 2013). We did not use any dimension reduction. We performed regression on the PSA matrices with a GLM including our main experimental regressors of interest, confound regressors, and two exploratory regressors of interest that are described in the supplement.
